# Percutaneous vs. Surgical Tracheostomy: A Comparative Analysis of Efficiency, Cost, and Hospital Stay

**DOI:** 10.7759/cureus.90492

**Published:** 2025-08-19

**Authors:** Rollin William Johnson, Antonio Melhem, Nischal Bogati, Oscar Rios Herrera, Kaleab Debebe, Hector DePaz, Leaque Ahmed, Mohammad Gilani

**Affiliations:** 1 General Surgery, Wyckoff Heights Medical Center, Brooklyn, USA; 2 Surgery, Wyckoff Heights Medical Center, Brooklyn, USA

**Keywords:** cost reduction, critical care outcome, open tracheostomy, percutaneous tracheostomy, surgical critical care

## Abstract

Introduction

Tracheostomy is a regularly performed procedure for patients requiring prolonged ventilatory support in the intensive care unit (ICU). Our community hospital implemented a new percutaneous tracheostomy (PT) program in an attempt to decrease operational costs and navigate operating room limitations. After the successful rollout, we performed a study comparing PT and surgical tracheostomy (ST) in terms of efficiency, ICU stay, and cost after one year to determine the outcomes a community hospital could expect in the early stages of PT implementation.

Methods

This is a cohort study with retrospective data collected from hospital records for both ST and PT over the years 2023-2024. Outcomes measured included consult-to-procedure time, procedural duration, ICU stay, and cost. Statistical significance was set at p<0.05.

Results

PT had a significantly shorter consult-to-procedure time (p=0.0039), with a mean of 1.76 days, compared to 4.139 days for ST. PT was also faster in procedural duration (p=0.0001), averaging 8.66 minutes, versus 53.93 minutes for ST. ICU length of stay showed no statistical difference (p=0.3919), with PT patients staying three days versus 3.79 days for ST. Cost analysis revealed PT was significantly more cost-effective, averaging $971.93, compared to $2,397.98 for ST.

Conclusion

PT is a more efficient and cost-effective alternative to ST, significantly reducing consult-to-procedure time and procedure duration. Although ICU length of stay did not reach statistical significance, PT demonstrates clear advantages in terms of resource utilization and cost savings.

## Introduction

Tracheostomy is a common procedure for critically ill patients requiring prolonged mechanical ventilation. It is integral to accelerating a patient's ventilation wean and speech rehabilitation [1]. The two main techniques, namely, percutaneous tracheostomy (PT) and surgical tracheostomy (ST), differ in approach, efficiency, and cost. ST has been the long-standing technique used to ventilate a patient through a surgically created tract, between the second and fourth laryngeal rings. Tracheostomy creation through PT is a technique that involves running dilators, and eventually a cannula, over a percutaneously placed guide wire [2]. PT has more recently gained popularity due to its bedside feasibility, reduced resource demands, and long-standing comparative safety to ST [3,4]. Picking the correct technique for ST is a patient-dependent decision; however, a recent literature review by our authors revealed studies supporting safety in previously contraindicated circumstances for PT [5-7]. Studies have even gone as far as to support PT over ST in the setting of coagulopathy, such as with Kim et al. who demonstrated better outcomes in cirrhotic patients [8]. With an increase in viability, the delineation of the strengths and weaknesses of the PT versus ST needs to be explored. Furthermore, this paper will examine the viability of PT within a community hospital and the ability of PT to overcome intrinsic limitations within this setting. While PT has been shown to be viable in large academic centers and community hospitals, some of the direct advantages of PT over ST have yet to be explored in a small hospital with a lack of resources [9,10]. This study evaluates key outcomes, including consult-to-procedure time, procedural duration, post-procedure intensive care unit (ICU) stay, and cost-effectiveness, to determine the potential advantages of PT over ST.

## Materials and methods

A retrospective cohort study was conducted at our institution, Wyckoff Heights Medical Center, located in Brooklyn, a small urban community hospital with limited resources, over the last two years, by reviewing 12 patients who underwent PT and 43 patients who underwent ST. The ST cohort's data were collected over two years retrospectively through review of charts, operative reports, and clinical notes, while the PT data were collected over a single year retrospectively in the same manner after the rollout of our pilot PT program. The decreased number of PT compared to ST is attributed to the early application of PT at our institution and careful patient selection. Both forms of tracheostomy creation were performed based on availability and call schedule by all of our General Surgery physicians, whose experience level ranged from third-year attending to the chair of surgery.

PT was performed with the Blue Rhino method, which has been shown as the superior methodology in meta-analysis [11]. Inclusion criteria included adult patients requiring tracheostomy for prolonged mechanical ventilation. Patients requiring emergent tracheostomies were excluded. All patients in both cohorts required tracheostomy due to prolonged intubation secondary to acute hypoxic respiratory failure. Of the patients who underwent PT, five (42%) were intubated for pneumonia, four (33%) for cardiac arrest, and three (25%) for stroke. The number of ST patients intubated for pneumonia was 17 (39%); for cardiac arrest, eight (18.6%); for stroke, seven (16.3%); for chronic obstructive pulmonary disease (COPD) exacerbation, five (11.6%); and for respiratory failure due to other comorbidities, six (13.9%). The chi-squared analysis of the cause of intubation between PT and ST confirmed no statistical difference between the groups (p=0.35). None of the patients had a tracheostomy performed for airway obstruction or stenosis. Additionally, any patients with relative or absolute contraindications to PT, as defined by Mehta and Mehta, did not undergo PT [12]. None of the patients in either the PT or the ST grouping was on therapeutic anticoagulation. Data were collected on consult-to-procedure time, procedural duration, post-procedure ICU stay, and total hospital cost as the primary outcomes of interest. The cost of the PT was calculated by adding the sum of the price of the equipment and medications used to perform the procedure, whereas the cost of ST was calculated by averaging the operative time of a ST and multiplying that number by the estimated cost per minute of operating room (OR) use, which was deemed to be $46 a minute [13]. Demographic data collected included age, sex, body mass index (BMI), international normalized ratio (INR), partial thromboplastin time (PTT), platelet count, total hospital days, and days ventilated before surgery. These values were then compared between the two groups to confirm homogeneity between the ST and PT groups.

Statistical analysis for consult-to-procedure time, procedure duration, and ICU stay was performed using the two-tailed t-test. The demographic data set did not uniformly follow a normal distribution and are presented as mean (SD) with comparison between groups using the Mann-Whitney U test. Categorical variables are presented as frequencies and percentages and compared using the chi-squared test. Statistical significance was defined as p<0.05, and confidence intervals (95% CI) were calculated for all outcomes. All statistics were calculated using R statistical software (R version 4.5.1, R Foundation for Statistical Computing, Vienna, Austria). 

## Results

**Figure 1:**
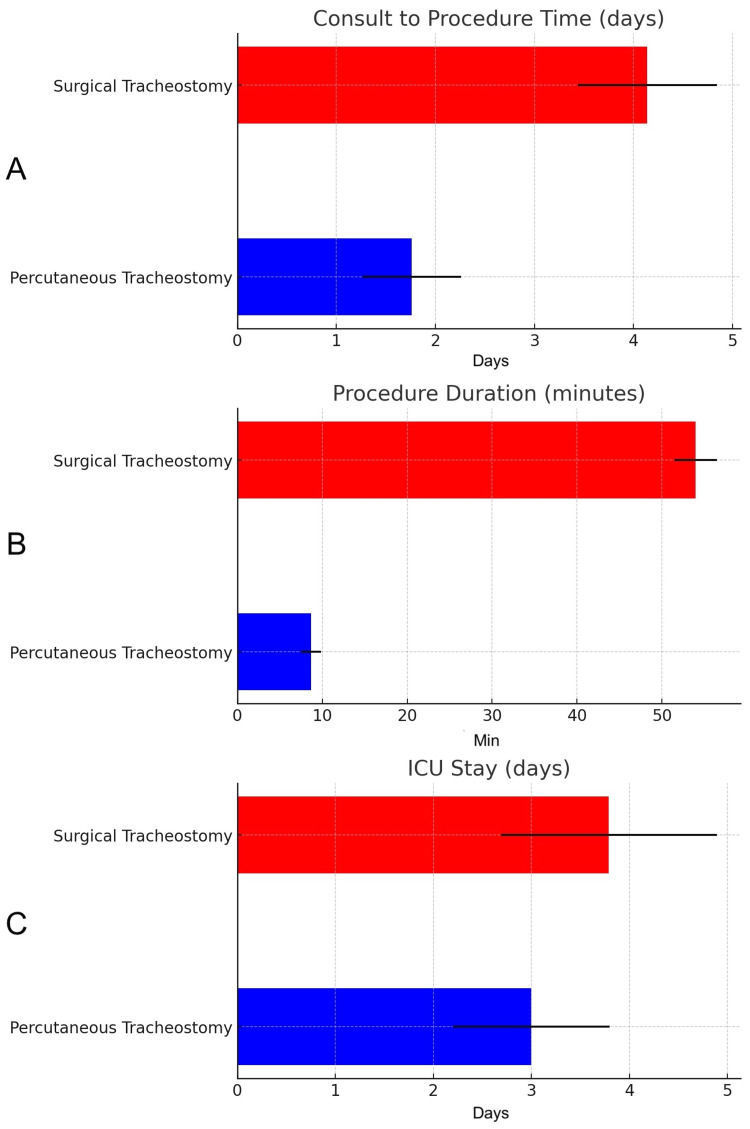
Comparison of primary outcomes between percutaneous and surgical tracheostomy All of the following p-values were calculated with the two-tailed t-test. (A) Consult-to-procedure time marked in days (p=0.0039). (B) Duration of the procedure in minutes (p=0.0001). (C) Total time in ICU marked in days (p=0.3919). Error bars represent standard deviations. ICU: intensive care unit

Patients undergoing PT had a significantly shorter consult-to-procedure time compared to ST (p=0.0039). The mean time for consult-to-procedure time was 1.76 days with a range of eight hours to seven days for PT and 4.139 days with a range of 1-12 days for ST, with a mean difference of -2.2929 days (95% CI: -3.8182 to -0.7675) (Figure 1A). PT was also significantly faster in terms of procedural duration (p=0.0001), with a mean duration of 8.66 minutes, compared to 53.93 minutes for ST, resulting in a mean difference of -45.27 minutes (95% CI: -56.45 to -34.09) (Figure 1B). Although ICU length of stay did not reach statistical significance (p=0.3919), PT patients had a mean ICU stay of three days, compared to 3.79 days for ST, with a mean difference of -0.79 days (95% CI: -2.63 to 1.05) (Figure 1C). All characteristics data showed no statistical significance between PT and ST, with the exception of days ventilated before surgery and the mean of PT being 9.9 days and the mean of ST being 12.69 (U=165; p=0.05) (Table 1). 

**Table 1:** Patient characteristics Male and female p-values were calculated with the chi-squared test and presented as a proportion (percent), and all other p-values in the table were calculated with the Mann-Whitney U test and presented as a mean (SD). All p-values of statistical significance were set to p<0.05. BMI: body mass index; INR: international normalized ratio; PTT: partial thromboplastin time

Characteristics	Percutaneous tracheostomy	Surgical tracheostomy	P-value
Age (years)	69.6 (80.5-58.7)	62.2 (77.9-46.5)	0.21
Male	6/12 (50%)	23/54 (43%)	0.83
Female	6/12 (50%)	31/54 (57%)	0.83
BMI (kg/m²)	25.1 (30.54-19.63)	30.7 (41.3-20.1)	0.14
INR	1.17 (1.39-1.01)	1.16 (1.31-1.00)	0.1
PTT	30.6 (36.8-24.5)	29.4 (35.4-23.4)	0.14
Platelets (k/uL)	208 (339-77)	261 (381-141)	0.11
Total hospital stay (days)	31.4 (42.6-20.3)	34.3 (47.3-21.3)	0.52
Days ventilated before surgery (days)	9.9 (13-6.8)	12.69 (17.5-7.88)	0.05

**Figure 2:**
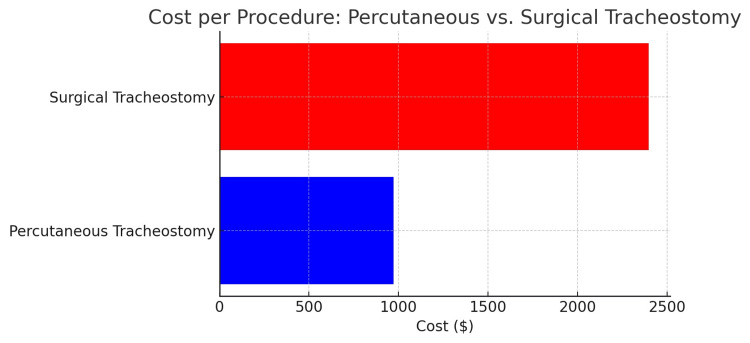
Cost per percutaneous vs. surgical tracheostomy Cost comparison of percutaneous versus surgical tracheostomy, highlighting the financial advantage of percutaneous tracheostomy due to reduced procedure time and avoidance of operating room expenses.

Cost analysis demonstrated that PT was substantially more cost-effective, with an average cost of $971.93, compared to $2,397.98 for ST (Figure 2). This cost difference was largely attributed to the avoidance of OR usage, which has an estimated cost of $46 per minute [13]. Additionally, the PT cohort lacked any complications. The ST cohort has a single postoperative bleed that required return to the OR.

## Discussion

The findings of this study indicate that PT offers substantial advantages over ST in terms of procedural efficiency, cost-effectiveness, and resource utilization. The significant reduction in consult-to-procedure time and operative duration highlights PT's role in expediting patient care, which is especially beneficial in high-demand hospital settings.

A major challenge associated with ST at our institution has been the delay in scheduling, often due to OR availability, lack of surgical staff starting in the late evening, and prioritization of emergent cases. The ability to perform PT at the bedside eliminates these logistical barriers, leading to a notable decrease in consult-to-procedure time. Following protocol modifications at our institution, some PTs were performed on the same day as consultation, further underscoring the efficiency of this approach. Although the difference in post-procedural ICU stay did not reach statistical significance in our study, we believe that with a larger sample size, this trend would become significant, which would be congruent with current literature [14,15]. This is an important causality to establish, as a decrease in ICU stay contributes to overall hospital efficiency and frees critical care resources.

Appropriate timing to perform tracheostomy among patients in the ICU remains controversial, with more authors recently advocating for early tracheostomy (<10 days) to reduce the length of ICU stay and associated morbidity and mortality. Vahapoğlu et al. found no effect of PT timing on 28-day mortality risk when comparing those who underwent PT before versus after a 14-day stay in the ICU [16]. The authors of this manuscript still encourage PT, tailored to the experience of the physician and the standard procedures in the ICU, as it has been shown to reduce the total duration of mechanical ventilation and length of ICU stay [14,17]. Interestingly, both of these values were not significant in our findings; however, we did have a statistically significant finding of a decrease in total hospital stay. 

One of the major successes of our pilot PT program was the lack of complications. This is likely attributed to the strict adherence to the exclusion of patients with relative contraindications to PT. This does highlight that a high level of safety can be achieved in early PT programs, which is further underscored in other papers [4]. Our findings have also outperformed on safety metrics compared to recent retrospective studies, likely due to stricter patient selection [18]. This is an encouraging finding for community hospitals looking to implement their own PT programs.

We took careful consideration to not include patients on therapeutic anticoagulation in our cohort. Surgeons advocating for ST have raised safety concerns related to potential post-intervention bleeding. Guo et al. investigated post-PT bleeding complications among neuro-critical care patients who are on anticoagulants or antithrombotics versus those with a normal coagulation profile. They found that PT is safe and feasible among patients receiving anticoagulant and/or antithrombotic therapy, with no significant increase in complications [5] (p>0.05). Even in the face of this data, it is still advised by our surgical group in the early period of a PT program to avoid performing PT on any patients with relative contraindications.

Financial considerations are another important factor in the adoption of PT. The study findings demonstrate that PT reduces costs by shortening procedure duration and eliminating OR expenses, which are estimated at $46 per minute [13]. The cost burden of ST stems primarily from the high cost of an OR per minute. Eliminating this expense from tracheostomy creation allows hospitals to reallocate funds to other critical services, ultimately improving overall healthcare delivery. Further financial efficiency can be seen in hospitals with limited OR availability but high case loads by allowing an ST time slot to be used for cases that would otherwise be scheduled for the next day [19]. Our study also found a decreased overall hospital stay, which has been associated with further cost savings [20]. 

There are limitations to this study that warrant further discussion. The clearest limitation is the retrospective nature of the study, which lacks randomization and blinding. The sample size of the PT cohort is also relatively small and likely prevented statistical significance from being achieved for the number of total ICU days, which has been seen to be reduced for PT in other studies [14,15]. The limited PT cohort size is due to data being from a single year and all PT being performed on patients without any relative contraindication to surgery. This also leads to an opportunity for selection bias; however, surgical morbidity and mortality were not of interest in this study, and so the primary objectives of this study were likely unaffected. Another limitation of this paper is the lack of comparison between ST's and PT's past medical history outside of the reason for intubation.

The implications of this study are particularly relevant for small community hospitals with limited OR availability. By integrating PT into routine practice, these facilities can offer timely tracheostomy placement, minimizing delays and reducing the risk of complications associated with prolonged intubation. Moreover, bedside PT enhances workflow efficiency, decreasing dependence on OR scheduling and alleviating bottlenecks in surgical services.

Despite these advantages, PT is not suitable for all patients. Anatomical considerations, coagulopathies, and operator expertise must be carefully evaluated before determining the appropriate approach. Further research, including multi-center studies with larger patient cohorts, is needed to strengthen the evidence base for PT and refine patient selection criteria.

## Conclusions

PT offers clear advantages over ST in terms of efficiency and cost-effectiveness. The significant reduction in consult-to-procedure time and procedural duration supports PT as a preferred approach in appropriate patient populations. While ICU stay differences were not statistically significant, PT remains a valuable option in critical care settings, offering both clinical and financial benefits. Given the resource constraints of small community hospitals with busy ORs, PT provides an effective solution for timely tracheostomy placement and improved patient management.
